# Ghostwriting: The Dirty Little Secret of Medical Publishing That Just Got Bigger

**DOI:** 10.1371/journal.pmed.1000156

**Published:** 2009-09-08

**Authors:** The PLoS Medicine Editors

## Abstract

In an academic_editorial highlighting the 1,500 documents made public after *PLoS Medicine*'s intervention in a court case, the *PLoS Medicine* academic_editors call for strong action to be taken to eradicate this practice.

If you are an editor, author, reviewer, or reader of medical journals, or if you depend on your doctor or health care provider getting unbiased information from medical journals, then the 1,500 documents now hosted on the *PLoS Medicine* Web site [1] should make you very concerned and angry. Because, quite simply, the story told in these documents amounts to one of the most compelling expositions ever seen of the systematic manipulation and abuse of scholarly publishing by the pharmaceutical industry and its commercial partners in their attempt to influence the health care decisions of physicians and the general public.

Here's just one sample thread [2] that gives an idea of the topsy-turvy world invented by the pharmaceutical and medical writing companies involved. While readers expect and assume that the named academic authors on a paper carried out the piece of work and then wrote up their article or review informed by their professional qualifications and expertise, instead we see a prime example of “ghostwriting”: a writing company was commissioned to produce a manuscript on a piece of research to fit the drug company's needs and *then* a person was identified to be the “author”:

An email from a writer employed by the medical writing company, DesignWrite, to employees of Wyeth, the company that performed the study, and Parthenon (another medical writing company) on November 10, 2003 concerning manuscripts on Totelle (a brand of hormone replacement therapy manufactured by Wyeth) tells the story concisely. “Thanks to all who have reviewed and approved the manuscripts… *I have received no word on authors for the Totelle 2 mg bone manuscript P3(2), and need input on this matter before this manuscript can move forwards.*” [our emphasis added]

*PLoS Medicine* became involved in this particular ghostwriting story when we intervened in an ongoing court case [1] in which women were suing Wyeth, the manufacturers of Prempro, a hormone replacement therapy. During the discovery process for this case, one of the lawyers representing injured women in the litigation, Jim Szaller of Cleveland, Ohio, became aware of many documents that laid out in detail the company's (mostly successful) attempts to publish papers written by unacknowledged professional medical writers in which the message, tone, and content had been determined by the company but the paper was subsequently nominally “authored” by respected academics—in sum a coordinated and carefully monitored campaign of ghostwriting. Our interest was not in the specific drugs, but in the issue of ghostwriting itself, a topic we have long been interested in and published on [3]–[6] The intervention, presented by lawyers from public interest law firm Public Justice (http://www.publicjustice.net), and a similar one from the *New York Times*, was successful. On July 24, 2009, US District Judge William R. Wilson, Jr., in Little Rock, Arkansas, granted the Motions of the Interveners, and the similar Motion of the lawyers representing the women, to make the discovery materials public as of July 31.

This is not the place to review everything written on this topic. Others have written about ghostwriting campaigns concerning single drugs that have led to catastrophic health effects [7], and how even research papers and clinical trials are affected by ghost authors [7],[8]. What's clear is that ghostwriting can no longer be considered one of the “dirty little secrets” of medical publishing that nothing can be done about. While editors, medical schools, and universities have turned a blind eye to, or at the least failed to tackle head-on the pervasive presence of ghostwriting, drug companies and medical education and communication companies have built a vast and profitable ghostwriting industry. Recruitment of academic “authors” appears, within some academic circles, to have come to be considered acceptable, and marketing campaigns are no longer orchestrated around paid display advertisements but instead center on “evidence” provided by seemingly respectable academic review articles, original research articles, and even reports of clinical trials. What, a cynical reader might ask, can I truly trust as being unbiased? The answer is that, sadly, for some or even many journal articles, we just don't know.

So what can be done? The documents that have been made available are a substantial step forward in advancing knowledge of this practice and explaining the mechanics of how ghostwriting campaigns are organized, and will add to the evidence base. By making them easily and openly accessible we hope that others will quickly delve into the documents and analyze them in detail (we have yet not done so in the interest of speed in making them publicly available). But we also hope that the papers not only will become the subject of academic scrutiny but will help to guide the way to identifying reforms that will eventually stamp ghostwriting out. In an environment in which drug companies are beholden to their shareholders, and the drive for profit takes center stage, it is naïve to think that companies will put their own houses in order.

Over the past several years some journals and editors' organizations [9],[10], and even some individual medical writers [11], have pursued what might be called a war of attrition against the practice by requiring contributorship statements for authors and publishing them, insisting on the naming of all who were involved in writing, requiring detailed competing interest statements, and detailing and publishing the provenance of non-research articles. Editors' bodies such as the International Committee of Medical Journal Editors (ICMJE) expressly define criteria for authorship in biomedical publications [12], and the World Association of Medical Editors (WAME) developed a specific policy on ghostwriting [10] initiated by commercial companies that calls the practice dishonest, unacceptable, and sanctionable. But it seems that these tactics are simply not enough to prevent ghostwriting, and are being sidestepped by those involved. Although medical writers can and do have a legitimate place in *assisting* in the preparation of manuscripts (and, of course, academics and pharmaceutical companies can have legitimate and appropriate relations, and not all papers in this archive will have been written by ghost authors), attempting to hide the presence of ghostwriters or the involvement of writers beyond technical support (such as copyediting) is unacceptable. We'd argue, therefore, that all involved must adopt a much tougher approach of complete nontolerance to practices that aim to conceal authors or where the involvement of medical writers goes beyond technical support.

What might this mean in practice for journals? Primarily, it would mean a sea change in the thinking and behavior of editors, who should create—and be prepared to enforce—journal policies clarifying that involvement with ghostwriting is a serious and punishable breach of publication ethics. Of course, prevention is key: possible measures could include requiring statements upon submission from academic authors about involvements by any company whose products are mentioned (positively or negatively, directly or indirectly) in the commissioning of a third party to provide editorial assistance, manuscript preparation, or submission of the paper.

But journal polices should also include enforceable sanctions. For example, if nothing is declared on submission but inappropriate involvement of a medical writer subsequently comes to light, any papers where this breach is substantiated should be immediately retracted and those authors found to have not declared such interest should be banned from any subsequent publication in the journal and their misconduct reported to their institutions.

In the case of the documents deposited here, a good start, and a signal of the seriousness of journals' intent, would be the formal retraction of all the papers mentioned in which ghostwriting has been conclusively shown. Institutions whose academics are shown to be involved should investigate as a matter of urgency.

It's time to get serious about tackling ghostwriting. As has been shown in the documents released after the Vioxx scandal [7], this practice can result in lasting injury and even deaths as a result of prescribers and patients being misinformed about risks. Without action, the practice will undoubtedly continue. How did we get to the point that falsifying the medical literature is acceptable? How did an industry whose products have contributed to astounding advances in global health over the past several decades come to accept such practices as the norm? Whatever the reasons, as the pipeline for new drugs dries up and companies increasingly scramble for an ever-diminishing proportion of the market in “me-too” drugs, the medical publishing and pharmaceutical industries and the medical academic community have become locked into a cycle of mutual dependency, in which truth and a lack of bias have come to be seen as optional extras. Medical journal editors need to decide whether they want to roll over and just join the marketing departments of pharmaceutical companies. Authors who put their names to such papers need to consider whether doing so is more important than having a medical literature that can be believed in. Politicians need to consider the harm done by an environment that incites companies into insane races for profit rather than for medical need. And companies need to consider whether the arms race they have started will in the end benefit anyone. After all, even drug company employees get sick; do they trust ghost authors?
